# The Interaction Between Microglia and Macroglia in Glaucoma

**DOI:** 10.3389/fnins.2021.610788

**Published:** 2021-05-28

**Authors:** Xiaohuan Zhao, Rou Sun, Xueting Luo, Feng Wang, Xiaodong Sun

**Affiliations:** ^1^Shanghai Key Laboratory of Ocular Fundus Diseases, Shanghai General Hospital, Shanghai Engineering Center for Visual Science and Photomedicine, Shanghai, China; ^2^Department of Ophthalmology, Ninth People’s Hospital, Shanghai Jiao Tong University School of Medicine, Shanghai, China; ^3^Department of Immunology and Microbiology, Shanghai General Hospital, The Center for Microbiota and Immunological Diseases, Shanghai Institute of Immunology, Shanghai Jiao Tong University School of Medicine, Shanghai, China

**Keywords:** microglia, macroglia, astrocytes, Müller cells, glaucoma, neuroinflammation

## Abstract

Glaucoma, a neurodegenerative disease that leads to irreversible vision loss, is characterized by progressive loss of retinal ganglion cells (RGCs) and optic axons. To date, elevated intraocular pressure (IOP) has been recognized as the main phenotypic factor associated with glaucoma. However, some patients with normal IOP also have glaucomatous visual impairment and RGC loss. Unfortunately, the underlying mechanisms behind such cases remain unclear. Recent studies have suggested that retinal glia play significant roles in the initiation and progression of glaucoma. Multiple types of glial cells are activated in glaucoma. Microglia, for example, act as critical mediators that orchestrate the progression of neuroinflammation through pro-inflammatory cytokines. In contrast, macroglia (astrocytes and Müller cells) participate in retinal inflammatory responses as modulators and contribute to neuroprotection through the secretion of neurotrophic factors. Notably, research results have indicated that intricate interactions between microglia and macroglia might provide potential therapeutic targets for the prevention and treatment of glaucoma. In this review, we examine the specific roles of microglia and macroglia in open-angle glaucoma, including glaucoma in animal models, and analyze the interaction between these two cell types. In addition, we discuss potential treatment options based on the relationship between glial cells and neurons.

## Background

Glaucoma is one of the main causes of irreversible vision and visual field loss. As a progressive optic nerve disorder, its main pathological manifestations are loss of retinal ganglion cells (RGCs) and degenerative lesions of the optic nerve (Weinreb et al., 2014; Gordon and Kass, 2018; Calkins, 2021). Elevated intraocular pressure (IOP) has been recognized as a key risk factor for glaucoma. Therefore, the main prevention and treatment measure for high-tension glaucoma is adequate control of IOP (Weinreb and Khaw, 2004; Weinreb et al., 2014; Conlon et al., 2017; Gordon and Kass, 2018). However, some patients with normal IOP also have glaucomatous visual impairment and RGC loss. This suggests that in addition to high IOP, there may be other factors that cause pathological glaucomatous changes. For example, the duplication of TBK1 [tumor necrosis factor (TNF) receptor-associated nuclear factor kappa-B (NF-κB) activator (TANK)-binding kinase 1], a gatekeeper of neuroinflammation (Ahmad et al., 2016), can be detected in hereditary normal tension glaucoma patients (Fingert et al., 2011). Wild-type mice could acquire glaucoma-like characteristics, such as loss of RGCs, by receiving T cells from Sh3pxd2b^nee^ mice, a hereditary glaucoma model with elevated IOP (Gramlich et al., 2015). This indicates that neuroinflammation and the immune system can initiate the occurrence and progression of glaucoma.

In glaucoma, the cells involved in immunoregulation in the retina mainly include microglia, astrocytes, and Müller cells, the latter two being macroglia (Adornetto et al., 2019; Reichenbach and Bringmann, 2020). In a normal retina, these cells provide nutrition and structural support, participate in metabolism, and regulate homeostasis while coordinating with each other in the regulation of the state of neurons through phagocytosis and the secretion of inflammatory cytokines and neurotrophic factors (Vecino et al., 2016). Thus, microglia and macroglia do not function in isolation, and a delicate balance always exists between them. Glial cells work together to maintain the stability of the retinal microenvironment. However, once there is a deviation in this delicate balance, effects opposite to what is expected may occur. Therefore, understanding the interaction between glial cells plays an essential role in better glaucoma prevention and treatment.

In this review, we discuss the role and importance of microglia and macroglia in open-angle glaucoma, including glaucoma in animal models. The animal models discussed in this article are RGC loss models, most of which are high IOP models; models with RGC loss caused by other reasons are also mentioned. We analyze the contribution of the interactions between microglia and macroglia to the survival of RGCs and optic nerves with the hopes of providing additional insights into the relationship between glial cells and neurons while also providing some foundations and directions for future research. Based on the functions and characteristics of different glial cells, we propose future research directions for glial cells for better prevention and control of glaucoma.

## Microglia in Glaucoma

Microglia, which are full-time phagocytes, play an important role in innate immune responses. Microglia are involved in neural circuits and angiogenesis in a developing retina, whereas they regulate retinal neuron activity and synaptic integrity in a mature retina (Silverman and Wong, 2018; Chen et al., 2019; Rathnasamy et al., 2019). In a healthy retina, perivascular microglia control the substances entering the retina from the circulatory system, whereas other scattered retinal microglia patrol the microenvironment to remove metabolites and cell debris and mediate synaptic remodeling (Provis et al., 1996). Microglia are distributed horizontally in the plexiform layer of the inner retina and make regular short-term contact with neuronal synapses at rest (Wake et al., 2009). They respond quickly and sensitively to pathological stimuli, such as lipopolysaccharides, complements, thrombins, inflammatory cytokines, and chemokines, migrating to an injury site within 24 h (Okunuki et al., 2018).

In normal human eyes, quiescent microglia, with thin ramified processes, are found around the optic nerve head (ONH) in the walls of large blood vessels and in surrounding capillaries in glial columns and the cribriform plate. In human glaucomatous eyes, microglia are activated as clusters of large ameboids in the compressed lamina cribrosa (LC) and as formations of concentric circles surrounding blood vessels. Microglia are also redistributed to the parapapillary chorioretinal region and are present as single cells or clusters at the termination of Bruch’s membrane (Neufeld, 1999). In the ONH of glaucomatous eyes, microglia express different cytokines and mediators in addition to their morphology and distribution. Research has shown that abundant transforming growth factor-β2 (TGF-β2), TNF-α, and proliferating cell nuclear antigens are present in the microglia of glaucomatous ONH, whereas no positive signal of these factors is detected in the microglia of normal ONH (Yuan and Neufeld, 2001).

Microglial activation is considered one of the early alterations that occur in glaucoma. For example, in chronic hereditary glaucoma model DBA/2J mice, the aggregation, activation, and redistribution of microglia can be detected before RGC injury (Bosco et al., 2011). In DBA/2 J mice, the transcriptome of ONH microglia changes significantly in the metabolic, phagocytosis, inflammatory, and sensome pathways (Tribble et al., 2020). Among these, microglial surveillance and phagocytosis are downregulated, whereas metabolism-related transcripts are upregulated. These findings suggest that chronic ocular hypertension inhibits the homeostasis and related functions of microglia, and that ONH microglia increase the capacity to metabolize energy (Tribble et al., 2020). Retinal microglial reactions are regulated by many factors, such as microRNAs (miRNAs). Previous investigations have indicated that miRNAs are associated with microglial activation and polarization (Yan et al., 2015; Drewry et al., 2018; Wang et al., 2021). In a study of an acute ocular hypertension rat model, overexpression of microRNA-93-5p reduced microglial proliferation, migration, and cytokine release; these findings were accompanied by reduced loss of RGCs (Wang et al., 2021).

Despite these reported findings, the role of microglia in glaucoma remains controversial. Some researchers have suggested that microglia can aggravate RGC damage and neurodegeneration in multiple ways, including through the release of inflammatory cytokines, such as TNF-α, interleukin 1β (IL-1β), IL-6, matrix metalloproteinases, Fas ligands, and reactive oxygen species (Hanisch and Kettenmann, 2007; Langmann, 2007). In a study of an S100B-induced glaucoma-like animal model, microglia were activated and increased in the optic nerve on day 14, and RGC apoptosis was observed. However, on day 21, the microglial response was not as prominent, whereas RGC damage was still present (Grotegut et al., 2020). Therefore, the deletion of genes that affect microglial activation or other normal functions can contribute to the survival of RGCs or the integrity of the optic nerve. Microglia express high levels of complement peptide C3a receptor 1 (C3ar1), which has been identified as a damaging neuroinflammatory factor. Deficiency in C3ar1 lowers the risk of retinal degeneration independent of IOP in DBA/2J mice (Harder et al., 2020). The neutralization or genetic deletion of TNF-α significantly reduces RGC loss in a glaucoma mouse model with angle closure induced *via* argon laser irradiation (Nakazawa et al., 2006). Deletion of the CD11b/CD18 gene, which mediates the recruitment and activation of leukocytes, inhibits the loss of RGCs and combats neurotoxicity (Nakazawa et al., 2006). Meanwhile, a deficiency in C-X3-C motif chemokine receptor 1 (CX3CR1), which is expressed by neurons, can inhibit microglial reactivity, aggravate neurotoxicity, and induce extensive damage to RGCs in an experimental mouse glaucoma model with transient elevation of IOP (Wang K. et al., 2014). Some drug interventions can also exert beneficial effects on RGC survival by inhibiting microglial reactivity. Minocycline, a drug that reduces microglial activation, improves the integrity of RGC axons and the function of the optic nerve in DBA/2J mice (Bosco et al., 2008). Intravitreal injection of macrophage inhibitory factor in a rat optic nerve axotomy model (Thanos et al., 1993) and high-dose irradiation of the head of a DBA/2J mouse (Bosco et al., 2012) inhibit the degradation of neurons and promote the development of axons. Furthermore, the proliferation and activation of microglia has shown a significant correlation with the severity of optic neuropathy in DBA/2J mice (Bosco et al., 2015).

However, some studies have suggested that microglia can protect RGCs from damage. Microglia help to prevent secondary retinal damage by phagocytosing damaged or dead RGCs in the later stages of optic nerve damage (Sierra et al., 2013). It is well known that DNA can act as danger-associated molecular patterns, inducing tissue toxicity and organ damage in a mouse glaucoma model with transient elevation of IOP (Supinski et al., 2020). As major phagocytes, microglia can prevent DNA release from dying cells through phagocytosis (Egensperger et al., 1996). Once activated, microglia can clear multiple apoptotic RGCs for at least 14 days after reaxotomy of the RGCs (Schuetz and Thanos, 2004); however, inappropriate phagocytosis by microglia can accelerate the loss of RGCs (Brown and Neher, 2014). Therefore, regulation of phagocytosis enables microglia to function better as guardians during retinal injury. In addition, microglia produce neurotrophic factors (Harada et al., 2002), remove excess glutamate (Basilico et al., 2019), and exert antioxidant effects through antioxidant enzymes in the cells (Vilhardt et al., 2017) when the retina is damaged.

In summary, microglia exert neuroprotective or neurotoxic effects in glaucomatous eyes, depending on the secreted factors. Microglia are activated in the early stages of glaucoma and rapidly migrate to an injury site. They stabilize the retinal microenvironment through phagocytosis and secretion of anti-inflammatory cytokines, thus protecting the optic nerve and RGCs. In the late stage of glaucoma, however, overactivated microglia produce pro-inflammatory cytokines, complement, and other toxic factors that directly affect RGC apoptosis. There is no doubt that microglia are an important target for the prevention of glaucoma, and due to their complexity, more accurate and precise intervention and regulation involving them will be a challenge in future research.

## Macroglia in Glaucoma

Macroglia, which mainly include astrocytes and Müller cells, possess similar transcription profiles and functions (Xue et al., 2011). Their cell bodies surround the axons of neurons and form glial sheaths around neurons that protect and buffer them. Müller cells span the entire retinal layer, whereas astrocytes are confined to the innermost layer of the retina. The difference between their distribution indicates that their monitoring and reacting areas are different; Müller cells are more global, whereas astrocytes are local. Their close contact with neurons also allows macroglia to sensitively perceive changes in the microenvironment, such as hypoxia, and respond in a timely manner. Macroglia transport most nutrients, wastes, ions, water, and other molecules between blood vessels and neurons (Reichenbach and Bringmann, 2020). In addition, they contribute to various glial homeostatic functions, such as the regulation of extracellular pH and potassium balance (Agte et al., 2011).

When the retina is stimulated or injured, macroglia can be activated and can express glial fibrillary acidic protein (GFAP) and other extracellular matrix signals through enlarged cell bodies and thickened foot processes (Varela and Hernandez, 1997; Tezel et al., 2003; Lukowski et al., 2019), a process known as retinal gliosis. In a human glaucomatous retina, astrocytes and Müller cells show a hypertrophic morphology with increased immunostaining of GFAP, which suggests that retinal gliosis is exhibited in glaucoma (Tezel et al., 2003).

### Astrocytes in Glaucoma

As the main component of glial cells in the central nervous system, astrocytes play a variety of essential roles, including the regulation of ion balance, metabolic supply and structural support, neurotransmitter transmission, and synaptic plasticity (Halassa et al., 2007). Astrocytes not only play the role of maintaining retinal homeostasis but also directly act on the pathophysiology of RGCs and the optic nerve through a variety of factors.

In clinical practice, optic disk cupping can be easily discernible in glaucomatous eyes. ONH cupping can be induced by structural changes in the LC, which is enriched with collagens (Elkington et al., 1990). Optic nerve axons in the LC are unmyelinated and surrounded by astrocytes (Shinozaki and Koizumi, 2021). Activated astrocytes have been found in the human glaucomatous retina (Minckler and Spaeth, 1981; Tezel et al., 2003). Recently, intravitreal injection of S100B, which is mainly expressed by astrocytes, was found to cause glaucoma-like neuronal degeneration in the retina and optic nerve (Grotegut et al., 2020).

In glaucoma, astrocytes change from a resting state to an activated state in a process known as astrogliosis and act as early responders to induce inflammatory responses. Astrocytes have been shown to mediate the activation of multiple inflammatory pathways, including TNF-α signaling, NF-κB activation, autophagy, and inflammasome-associated regulators in high IOP rats after administration of hypertonic saline injections into their episcleral veins (Tezel et al., 2012). In patients with glaucoma and glaucoma animal models, astrocytes at the ONH can be observed to upregulate the expression of tenascin-C (Pena et al., 1999; Howell et al., 2011; Johnson et al., 2011), playing a pro-inflammatory role through the Toll-like receptor (TLR) 4 signaling pathway (Midwood et al., 2009). Astrocytes also induce the adhesion and migration of immune cells by secreting cell adhesion proteins (Johnson et al., 2011; Tanigami et al., 2012). They participate in the remodeling of the extracellular matrix of the LC, which is related to a variety of molecular pathways, including those for TGF-β, endothelins, bone morphogenetic proteins, and gremlin (Schneider and Fuchshofer, 2016).

In response to an increase in IOP, astrocytes expand and redistribute metabolites, donating resources from unstressed projections to stressed projections in DBA/2J mice (Cooper et al., 2020). Furthermore, once IOP is increased, activated astrocytes accompanied by upregulated cytoskeletal protein concentrate and tightly surround the ONH; however, long-term high IOP causes the astrocytes around the optic nerve to decrease or even disappear after magnetic microsphere injection (Dai et al., 2012). In the early stage of glaucoma progression in DBA/2J mice models, astrocytes become more parallel with migration to the edge of the nerve; however, the astrocytes’ parallelism diminishes as axons degenerate and glial coverage increases (Cooper et al., 2018). Moreover, some studies have shown that ONH astrocytes play a role in the engulfment of optic axons and promotion of axon degradation, which is one of the possible mechanisms for the sectorial nature of RGC loss in glaucoma (Nguyen et al., 2011; Mills et al., 2015).

Astrogliosis is beneficial to the retina, and its morphological remodeling is reversible in the early stages of the disease. However, in the late stage, it can cause excessive scar hyperplasia and degrade optic axons, which are detrimental to the repair of the body (Sun et al., 2013).

### Müller Cells in Glaucoma

Müller cells are the most abundant glial cells in the retina and are closely related to RGCs in anatomy and structure. They play a crucial role in neuronal development, survival, and related information processing (Bringmann et al., 2006). The cell body of a Müller cell is located in the inner nuclear layer, and its processes extend to the inner and outer layers of the retina. Müller cells tightly contact synapses and blood vessels in the inner and outer plexiform layers of the retina. Müller cells are rich in different ion channels, ligand receptors, transmembrane transport molecules, and enzymes (Bringmann et al., 2006). Their structural richness determines the diversity of their functions. Regarding physiological conditions, Müller cells are involved in retinal glucose metabolism, regulation of retinal blood flow and neurotransmitter transmission, and regulation of the balance of ions, water, and amino acids (Bringmann et al., 2006). Regarding pathological conditions, Müller cells regulate immunity and phagocytic cells or foreign substances. In particular, Müller cells undergo gliosis to cope with various types of retinopathy (Harada et al., 2002).

Müller cell reactions are detectable in the human glaucomatous retina through immunohistochemistry (Tezel et al., 2003). Müller cells regulate intracellular and extracellular glutamate levels through the expression of glutamate/aspartate transporter (GLAST) (Rauen, 2000). A study of GLAST knockout mice indicated that they showed spontaneous RGC loss and glaucomatous optic nerve degeneration without elevated IOP (Harada et al., 2007).

Müller cell gliosis mainly plays a protective role in the early stages of glaucoma. Investigation using scanning force microscopy has shown that Müller cells have very soft and flexible bodies with strong plasticity and powerful compliance (Lu et al., 2006). Furthermore, their hypertrophic cell bodies and increased intermediate filaments, such as GFAP, enhance resistance to mechanical forces, including elevated IOP (Lu et al., 2006). The expression of neurotrophins and their receptors can be detected in Müller cells after retinal ischemia–reperfusion (Vecino et al., 1998). In addition, Müller cells secrete neurotrophic factors, such as ciliary neurotrophic factor (CNTF), and the antioxidants glutathione (Pease et al., 2009) and ghrelin (Zhu et al., 2017), protecting RGCs from damage in glaucoma.

In the later stages of glaucoma, however, over-gliosis of Müller cells is dysfunctional, leading to disturbances in retinal homeostasis and neuronal death. Müller cells show excitotoxic damage to RGCs because their degradation of glutamic acid is weakened during hypoxia (Evangelho et al., 2019). Owing to the damage of the Müller cell membrane under high pressure, K^+^ siphoning is impaired, inducing cation dyshomeostasis in the microbead occlusion model (Fischer et al., 2019). They are not sufficient to rebalance microenvironmental changes and even activate neuronal pro-apoptotic pathways. Müller cell activation also aggravates the damage of RGCs through NF-κB-dependent TNF-α production (Lebrun-Julien et al., 2009), indicating that Müller cells indirectly contribute to neuroinflammation.

## Interaction Between Macroglia and Microglia in Glaucoma

**FIGURE 1:**
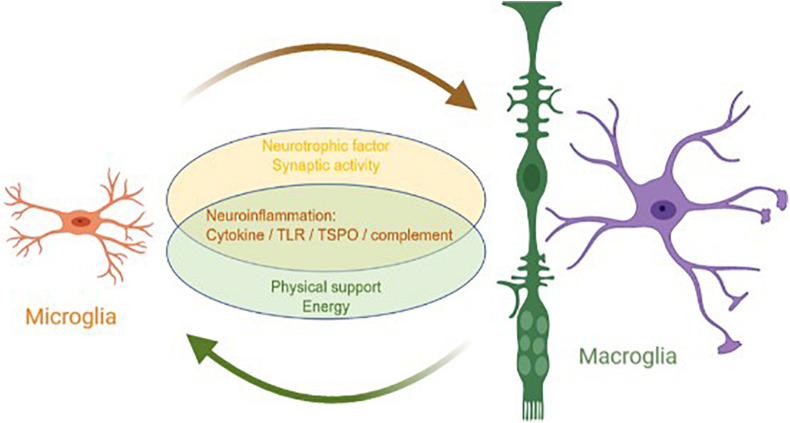
Interaction between macroglia and microglia in glaucoma. Microglia and macroglia are involved in the progression of neuroinflammation in glaucoma, including the inflammatory cytokine, TLR, TSPO, and complement pathways. In addition, microglia prompt macroglia to produce neurotrophic factors and help regulate synaptic activity, whereas macroglia provide microglia with the physical scaffold support and energy required for activities.

Although the origins and main functions of microglia and macroglia are different, they coordinate with each other to complete the nutrition, support, and protection of neurons (Figure 1). In a mature retina, macroglia, microglia, and pericytes inhibit the proliferation of endothelial cells and maintain the stability of blood vessels and the inner blood–retinal barrier (Gardner et al., 1997; West et al., 2005; Klaassen et al., 2013). In a healthy retina, the state of microglial activation depends directly on extracellular adenosine triphosphate (ATP). The main source of ATP is Müller cells, which means that Müller cells can indirectly serve as an energy source for microglia (Stout et al., 2002; Pankratov et al., 2006; Uckermann et al., 2006; Li et al., 2012).

After the retina is mechanically damaged or becomes degenerative, the interaction between microglia and macroglia begins immediately, and then the timing and extent of microglial activation is closely regulated (Wang M. et al., 2014). There are some similarities in the changes that occur in microglia and macroglia in glaucoma. In a study of IOP-independent experimental autoimmune glaucoma (EAG) mice models, the mice were immunized with an optic nerve antigen (ONA). Compared with the control group, the EAG mice displayed fewer Brna+ RGCs and SMI-31+ optic nerve neurofilaments, with a significantly higher number of retinal Iba1 + microglia and GFAP + astrocytes (Wiemann et al., 2020).

In a resting state, microglia mainly exhibit horizontal movement. However, with retinal injury or other stress, they begin vertical transretinal movement, which is closer to the behavior of Müller cells. Injection of lipopolysaccharides into the vitreous cavity of mice has shown that the horizontal branches of the microglia are in close contact with the cell bodies of Müller cells, suggesting that microglia may use Müller cells as scaffolds for transretinal migration (Sánchez-López et al., 2004; Wang et al., 2011). Müller cells co-cultured with activated microglia show more elongated spindles and multipolar shapes than their flat, thick lamellipodia when cultured alone (Wang and Wong, 2014). In addition, under stress, gliosis in retinal glia can be triggered by microglial activation through increased cytokine levels (Dharmarajan et al., 2017). Moreover, IL-6 (Fischer et al., 2014; Zhao et al., 2014) and interferon-γ (IFN-γ) (Cotinet et al., 1997) cause morphological changes and promote the production of inflammatory factors in Müller cells. In zebrafish, the pharmacological ablation of microglia results in a lack of reactivity in Müller cells with less GFAP expression; however, it does not affect the migration of Müller cells (Conedera et al., 2019). It has also been found that progesterone can reduce Müller cell gliosis by inhibiting the expression of IFN-γ from microglia in a retinitis pigmentosa mouse model (Roche et al., 2018). Activated microglia also regulate Müller cells to produce and release more trophic factors, such as basic fibroblast growth factor (bFGF) (Harada et al., 2002), glial-derived neurotrophic factor (GDNF), and leukemia inhibitory factor (LIF), thereby amplifying the protective effects of Müller cells. This response is independent of the expression of the typical glial markers of Müller cells (Pease et al., 2009). Activated microglia also participate in the maintenance of the synaptic activity microenvironment of macroglia, and they jointly regulate the content of ions and neurotransmitters (Kimelberg, 2010; Chong and Martin, 2015; Ma et al., 2019). Glial cells also contribute to the activation of the complement pathway in glaucoma (Pauly et al., 2019). The level of complements in Müller cells and microglia increases with transient retinal ischemia (Pauly et al., 2019). In addition, astrocytes activate C3 convertase, thereby amplifying complement signals and alerting microglia to respond to RGC damage (Howell et al., 2011; Astafurov et al., 2014; Williams et al., 2017).

Traditionally, microglia and astrocytes are considered to have two different phenotypes. Microglia are classified into “M1” and “M2” phenotypes. Simply put, M1 is an inflammation-promoting phenotype, whereas M2 is an anti-inflammatory phenotype (Varnum and Ikezu, 2012; Tang and Le, 2016). In animal models of laser-induced glaucoma, most microglial cells exhibit the M1 phenotype (Rojas et al., 2014). Similarly, astrocytes are categorized into “A1” and “A2” phenotypes. The A1 phenotype aggravates neuroinflammation, whereas A2 is neuroprotective (Liddelow et al., 2017). M1 and M2 microglia can stimulate reactive astrocyte changes by secreting different cytokines (Wei et al., 2019). M1 microglia, which secrete pro-inflammatory cytokines, can promote astrocytes to switch to the A1 phenotype, thereby increasing neurotoxicity and inducing neuronal death (Liddelow et al., 2017). Recently, single-cell RNA sequencing, which provides an unbiased analysis of cells, has been used to uncover more information about the heterogeneity of astrocytes and microglia. In studies of experimental autoimmune encephalomyelitis (EAE), astrocytes were identified as several subpopulations, and pro-inflammatory and neurotoxic subpopulations were the most expanded (Linnerbauer et al., 2020; Wheeler et al., 2020). Microglia show a variable spatial and temporal distribution during development and can gain a discrete context- and time-dependent disease-specific signature in the central nervous system pathology of mice. Corresponding clusters of microglia have also been identified in the brains of patients with multiple sclerosis (Masuda et al., 2019).

In addition to the aforementioned unidirectional coordination between glial cells, microglia and Müller cells are likely to have bidirectional feedback signals in response to injury or other stress. For example, activated microglia induce Müller cells to secrete more adhesion molecules and chemokines so that more microglia can adhere to the Müller cells and express higher chemotaxis, which also promotes microglial migration and recruits other immune cells to the injured areas (Evangelho et al., 2019). The upregulation of TLR signals in both microglia and astrocytes has been found in the retina of patients with glaucoma (Luo et al., 2010), and together they may mediate the initial immunity initiated by glaucoma stimulation signals. Both phagocytic microglia (Yuan and Neufeld, 2001) and reactive astrocytes (Zhang et al., 2004) express metalloproteinases, which increase during optic neuropathy, altering the amount of extracellular matrix to mediate retinal degeneration. Translocator protein (TSPO) and its ligand XBD173 (Mages et al., 2019) may be key mediators of the inflammatory response between microglia and macroglia (Scholz et al., 2015; Cooper et al., 2020). As initiators and amplifiers of retinal inflammation, activated microglia and Müller cells (Scholz et al., 2015) can mutually and reciprocally promote each other to produce more inflammatory cytokines, thereby creating a positive feedback loop and exacerbating the inflammatory response (Wang et al., 2011).

Generally, microglia are the main executors of neuroinflammation in a glaucomatous retina, whereas macroglia are supporters. Both microglia and macroglia undergo changes in morphology and physical location when the retina is under stress. As the only antigen-presenting cells of the retina, microglia are activated when an injury occurs and quickly migrate to the injury site. As the main supporting cells of the retina, macroglia also become mobilized and move parallel to the edge of the nerve, redistributing resources in response to the injury. These two types of glial cells coordinate to mediate neuroinflammation, including neuroinflammation from the cytokine, TLR, TSPO, and complement signaling pathways. Microglia are mainly involved in neuroinflammation, whereas macroglia regulate neuroinflammation in various ways. Furthermore, microglia prompt macroglia to produce neurotrophic factors and help regulate synaptic activity, whereas macroglia provide microglia with the physical scaffold support and ATP required for activities.

## Glia-Targeted Therapeutic Approaches

Overactivation of microglia is considered detrimental to the survival of RGCs. Therefore, protecting RGC by inhibiting the activity of microglia is an effective treatment for glaucoma. Some drug interventions exert beneficial effects on RGC survival by blocking microglial-relevant pathways. Microglia inhibitors have been shown to effectively protect RGCs in animal models (Drewry et al., 2018; Grotegut et al., 2020; Supinski et al., 2020). At present, microglia inhibitors have not been applied to the treatment of glaucoma. However, they have been used for the management of other diseases caused by retinal inflammation, an important aggravating factor for glaucoma. Treatment with the microglial inhibitory agent minocycline is associated with improved visual function, central macular edema, and vascular leakage, targeting the inflammatory etiology of diabetic macular edema in humans (Cukras et al., 2012).

In recent studies, macroglia have shown the potential for reprogramming and re-differentiation, which brings new hope for the treatment of retinal diseases. Glial cells and neurons are derived from the same pluripotent stem cells, and the influence of extracellular signals on them leads to different outcomes (Janowska et al., 2019). In theory, by regulating the extracellular signals of glial cells, their fate can be altered, and they can re-differentiate into glial cells or neurons. Targeting glia for neurorestorative therapy is a safe, effective, and efficient strategy. In the early stage of glaucoma, astrocytes and Müller cells limit the spread of inflammation and the progression of glaucoma. In the late stage of the disease, the overactive glial cells would form glial scars, which aggravate the progression of glaucoma (Reichenbach and Bringmann, 2020). Judging and selecting the appropriate intervention period may be the direction for the exploration of the astrocyte-based treatment in the future. In zebrafish, Müller cells enter a reprogramming state relatively easily, whereas in mammals, they enter a reactive state after injury; however, they stagnate before becoming progenitor cells (Hoang et al., 2020). How to regulate the entrance of mammalian Müller cells into the reprogramming state will be a future research direction.

## Conclusion

Many studies of patients with glaucoma and glaucoma animal models have shown that optic nerve damage or high IOP can cause the production of inflammatory cytokines and chemokines, as well as the activation of ocular immune cells, such as microglia and macroglia. A delicate balance between glial cells is critical for the development and maturation of the retina under normal conditions and during the pathological process of disease conditions. The neuroinflammation mediated by these cells is one of the key factors in the onset and progression of glaucoma. There is no doubt that IOP reduction is an effective prevention and treatment measure for patients with high-tension glaucoma. However, the roles of controlled neuroinflammation and promotion of neurotrophy in the treatment of glaucoma are also worth considering. However, microglia and macroglia do not function in isolation in glaucoma. Furthermore, an understanding of the interactions between glial cells is necessary for the development of new interventions to reduce neuroinflammation and prevent glaucoma. More research is needed to better understand the complex molecular spatiotemporal regulatory network in glaucoma. Further elucidation of the mechanism and response of glial cells in glaucoma will have a significant impact on the development of new cell-based therapies for retinal diseases.

## Author Contributions

All authors read and approved the final manuscript.

## Conflict of Interest

The authors declare that the research was conducted in the absence of any commercial or financial relationships that could be construed as a potential conflict of interest.

## Abbreviations

ATP: adenosine triphosphate
bFGF: basic fibroblast growth factor
CNTF: ciliary neurotrophic factor
Cx3cr1: C-X 3-C motif chemokine receptor 1
C3ar1: C3a receptor 1
EAE: experimental autoimmune encephalomyelitis
EAG: experimental autoimmune glaucoma
GDNF: glial-derived neurotrophic factor
GFAP: glial fibrillary acidic protein
GLAST: glutamate/aspartate transporter
IL-1 β: interleukin 1 β
IL-6: interleukin 6
IOP: intraocular pressure
LC: lamina cribrosa
LIF: leukemia inhibitory factor
MiRNAs: microRNAs
NF- κ B: nuclear factor kappa-B
ONA: optic nerve antigen
ONH: optic nerve head
RGCs: retinal ganglion cells
TANK: TNF receptor-associated factor NF- κ B activator
TBK1: TANK-binding kinase 1
TGF- β: transforming growth factor- β
TGF- β 2: transforming growth factor- β 2;
TLR: Toll-like receptor
TNF α: tumor necrosis factor α
TSPO: translocator protein.
